# *Candida tropicalis* Systemic Infection Redirects Leukocyte Infiltration to the Kidneys Attenuating Encephalomyelitis

**DOI:** 10.3390/jof7090757

**Published:** 2021-09-14

**Authors:** Natália Munhoz-Alves, Luiza Ayumi Nishiyama Mimura, Rosa Marlene Viero, Eduardo Bagagli, Jean Pierre Schatzmann Peron, Alexandrina Sartori, Thais Fernanda de Campos Fraga-Silva

**Affiliations:** 1 Department of Chemistry and Biological Sciences, Institute of Biosciences, São Paulo State University (UNESP), Botucatu 18618-689, Brazil; natalia.mnhz@gmail.com (N.M.-A.); luizamimura@gmail.com (L.A.N.M.); eduardo.bagagli@unesp.br (E.B.); alexandrina.sartori@unesp.br (A.S.); 2 Department of Pathology, Botucatu Medical School, São Paulo State University (UNESP), Botucatu 18618-687, Brazil; rosa.viero@unesp.br; 3 Neuroimmune Interactions Laboratory, Department of Immunology, Institute of Biomedical Sciences (ICB) IV, University of São Paulo (USP), São Paulo 05508-000, Brazil; jeanpierre@usp.br; 4 Postgraduate Program in Tropical Diseases, Botucatu Medical School, São Paulo State University (UNESP), Botucatu 18618-687, Brazil

**Keywords:** *Candida* spp., *Candida tropicalis*, autoimmunity, multiple sclerosis

## Abstract

Environmental factors, including infections, are strongly associated with the pathogenesis of multiple sclerosis (MS), which is an autoimmune and demyelinating disease of the central nervous system (CNS). Although classically associated with bacterial and viral agents, fungal species have also been suspected to affect the course of the disease. *Candida tropicalis* is an opportunistic fungus that affects immunocompromised individuals and is also able to spread to vital organs. As *C. tropicalis* has been increasingly isolated from systemic infections, we aimed to evaluate the effect of this fungus on experimental autoimmune encephalomyelitis (EAE), a murine model to study MS. For this, EAE was induced in female C57BL/6 mice 3 days after infection with 10^6^ viable *C. tropicalis* yeasts. The infection decreased EAE prevalence and severity, confirmed by the less inflammatory infiltrate and less demyelization in the lumbar spinal cord. Despite this, *C. tropicalis* infection associated with EAE results in the death of some animals and increased urea and creatinine serum levels. The kidneys of EAE-infected mice showed higher fungal load associated with increased leukocyte infiltration (CD45^+^ cells) and higher expression of T-box transcription factor (*Tbx21*) and forkhead box P3 (*Foxp3*). Altogether, our results demonstrate that although *C. tropicalis* infection reduces the prevalence and severity of EAE, partially due to the sequestration of leukocytes by the inflamed renal tissue, this effect is associated with a poor disease outcome.

## 1. Introduction

The genus *Candida* is composed of pleomorphic fungi that colonize the human gastrointestinal and genitourinary tracts. Although these fungi are found in their yeast form in the majority of healthy humans, they can also act as pathogens, especially in immunosuppressed individuals [1,2]. In general, most invasive forms of candidiasis manifest themselves as candidemia but can also infect the abdomen, lungs, bones, kidneys, central nervous system (CNS), and heart [3]. Several species of *Candida* can cause infections, being *C. albicans* the most prevalent species in humans [4,5]. However, *C. glabrata*, *C. parapsilosis*, *C. tropicalis*, and *C. krusei*, which are all known as non-*albicans Candida* species, have also been isolated in invasive infections [6], mainly from renal transplant recipients [7] and candidemia caused by these non-*albicans Candida* species was associated with underlying conditions such as chronic kidney disease [8]. In this regard, *C. tropicalis* was isolated from systemic infections all over the world, being the second most prevalent yeast in Brazil and the third in South America [9,10,11,12]. Moreover, *C. tropicalis* was reported in CNS infections [13,14] and associated to renal microabscesses [15,16].

Multiple sclerosis (MS) is an inflammatory, autoimmune and demyelinating disease of the CNS whose immunopathogenesis is extensively investigated by using the animal model denominated experimental autoimmune encephalomyelitis (EAE) [17,18]. Both MS and EAE are characterized by an immune response mediated primarily by CNS myelin-specific T lymphocytes, which determine gliosis, peripheral leukocyte infiltration, myelin sheath damage, and subsequent neuronal death [19]. It is accepted that this disease is triggered by a complex interaction between genetic and environmental factors. A large number of pathogens, including fungi, have been associated with MS [20]. From this standpoint, previous studies revealed high levels of anti-*Candida* antibodies and antigens associated to *C. albicans*, *C. famata*, *C. glabrata*, *C. parapsilosis*, and *C. tropicalis* in the cerebrospinal fluid from MS patients [21,22,23]. More recently, correlations have been described between *Candida* infection and MS progression [20]. Based on the evaluation of the expanded disability status scale, these authors observed decreased disease progression after treatment of MS patients with antifungal drugs.

Different hypotheses have been formulated to explain the possible participation of certain fungi groups in MS, such as the cross-reactivity of the fungi with human tissues, including CNS-related self-antigens [24], production of toxins that could release autoantigens through their toxic effects on astrocytes and oligodendrocytes [25] and the pro-inflammatory effect of the fungi in the CNS with microglia activation [26]. In this context, we demonstrated that the injection of gliotoxin, secreted by *Aspergillus fumigatus*, and the infection with viable yeasts of *C. albicans* aggravated the development of EAE, possibly through local induction of encephalitogenic cytokines [27,28]. Additionally, we showed that some non-*albicans Candida* species, including *C. glabrata*, *C. krusei* and *C. parapsilosis*, were able to disseminate to the CNS of both immunocompetent or immunosuppressed C57BL/6 mice during systemic infection and interacted in distinct ways with a microglia cell lineage [29].

The possible contribution of *C. tropicalis* to the development of MS has not yet been investigated neither in patients nor in its corresponding experimental model. Literature data suggest, however, that this species could potentially participate in this pathology. In this regard, it has been reported that *Candida* spp. infections can cause meningitis in immunosuppressed patients and in neurosurgical patients [30,31,32]. Moreover, *C. tropicalis* has been recently described as a cause of meningitis in humans [30] and was present, even though in a small percentage, in the oral cavity of MS patients [31]. Several virulence factors have been characterized in *C. tropicalis* strains, such as biofilm and hyphal formation, which can trigger oxidative stress responses [33]. In mice, *C. tropicalis* intravenous infection resulted in a progressive infection in the kidneys and transient multiplication in the brain [34]. These data suggest that the presence of *C. tropicalis* in the CNS could interfere with the local immune response. Thus, the current study was undertaken to evaluate whether the *C. tropicalis* infection reaches the CNS and interferes in the EAE development.

## 2. Materials and Methods

### 2.1. Experimental Design

To evaluate parameters related to the infection itself, mice were allocated into 3 experimental groups: CTL, non-infected control; *C. tropicalis* (3 days), and *C. tropicalis* (19 days). The last 2 groups correspond to the animals that were analyzed on the 3rd and 19th days after infection. The following assays were performed in these animals: body weight variation, fungal loads at the spleen, CNS, and kidneys, presence of inflammatory lesions and fungus at the spinal cord and kidneys, and cytokine production by cells from the spleen and kidneys in vitro stimulated with particulate *C. tropicalis* antigen. Then, to evaluate parameters concerning the effect of *C. tropicalis* infection on EAE development, 2 experimental groups were employed: EAE, mice that were submitted to encephalomyelitis induction; EAE/*C. tropicalis*, which were submitted to encephalomyelitis development 3 days after infection. The following evaluations were performed on the 16th day after EAE induction, that was, 19 days after fungus infection: clinical score, body weight variation, disease prevalence, survival, fungal load at the spleen, CNS, and kidneys, serum levels of urea, creatinine, albumin and total protein, inflammation and demyelination at the lumbar spinal cord, cytokine production by spleen cells in vitro stimulated with MOG neuroantigen and particulate *C. tropicalis* antigen, and characterization of leukocytes eluted from the CNS and kidneys by flow cytometry and RT-PCR. For sample collection, cardiac punction was performed in mice under deep terminal anesthesia (ketamine/xylazine), and then animals were perfused with 5 mL of sterile PBS.

### 2.2. Animals

Female C57BL/6 mice at 6 weeks old were purchased from a specific pathogen-free facility at the University of São Paulo (USP, Ribeirão Preto, SP, Brazil). The animals were kept in a sterile environment, in mini-isolators (Alesco, Monte Mor, SP, Brazil) containing a maximum of 5 mice per cage, with feed and water ad libitum. The experiments were started with 8–9 weeks old mice, and the biological samples were collected on the 16th day after EAE induction, that was, 19 days after infection. Animals were individually weighted daily to assess body weight. All procedures were performed in accordance with the local Ethics Committee on the Use of Animals from São Paulo State University (UNESP, Botucatu, SP, Brazil, protocol number 853).

### 2.3. Candida tropicalis Strain and Inoculum

The strain of *C. tropicalis* used in this study was originally isolated from a blood sample from a patient admitted to the Universitary Hospital of Botucatu (UNESP, Botucatu, SP, Brazil). This fungal isolate was initially characterized by Vitek and Chromagar, and speciation was further confirmed by the determination of rDNA internal transcribed spacer regions (ITS) and Maldi-Tof. This sample was labeled as H-2747 (Genbank Accession KX774383.1) and is now stocked in a collection at the Department of Chemistry and Biological Sciences (UNESP, Botucatu, SP, Brazil). The strain was kept frozen in 10% glycerol-yeast extract-peptone medium. For experimental use, the yeasts were grown in Sabouraud-dextrose agar (Becton Dickinson and Company, Sparks, MD, USA) plates for 24 h at 37 °C, then carefully washed and resuspended in phosphate-buffered saline (PBS). The fungal concentration was adjusted, by using cotton blue staining, to 1 × 10^7^ viable yeasts/mL in PBS. Animals were inoculated with 0.1 mL (1 × 10^6^ viable yeasts/animal) into the lateral tail vein.

### 2.4. EAE Induction and Clinical Evaluation

EAE was induced by subcutaneous immunization with 100 µg of myelin oligodendrocyte glycoprotein (MOG_35–55_) peptide (Genemed Synthesis Inc., San Antonio, TX, USA) emulsified in 25 µL of Complete Freund’s Adjuvant (Sigma-Aldrich, St. Louis, MO, USA) containing 4 mg/mL of *Mycobacterium tuberculosis* (Difco, Detroit, MI, USA). Mice also received 2 intraperitoneal doses (150 µg each) of *Bordetella pertussis* toxin (Sigma-Aldrich); one following immunization and the other 48 h later. EAE clinical severity was evaluated daily by body weight loss and clinical score determinations. The following score system was adopted: 0—no symptoms; 1—limp tail; 2—loss of hip tone; 3—partial hind leg paralysis; and 4—complete hind leg paralysis. The percentage of weight loss and the maximum clinical score was calculated considering the highest values observed in all animals during the experiment.

### 2.5. Serum Biochemical Analysis

To assess renal function, serum levels of urea, creatinine, total protein, and albumin were quantified with Bioclin commercial kits (Quibasa Química Basic, Belo Horizonte, MG, Brazil). Results were measured using a Cobas Mira Plus Chemistry Analyzer (Roche Diagnostic Systems, Seattle, WA, USA).

### 2.6. Fungal Load Determination

Samples from the spleen, kidneys, and CNS (brain and cervical spinal cord) were collected, weighted, and macerated, with the help of a plastic pestle for 1.5 mL microtube, in 1 mL of sterile PBS. A 100 µL aliquot from these homogenates was plated on Sabouraud-dextrose agar (Becton Dickinson and Company) plates and incubated for 3 days at 37 °C. After incubation, the number of colony-forming units (CFU) was counted, and the result was normalized per gram of tissue and logarithmized (log10).

### 2.7. Splenic and Renal Cell Cultures

Cells were obtained by maceration of spleen and kidneys and resuspended in Roswell Park Memorial Institute (RPMI) medium (Sigma-Aldrich) supplemented with 10% fetal bovine serum (Gibco, Invitrogen, Waltham, MA, USA), 1% L-glutamine, 1% sodium pyruvate, 1% nonessential amino acids, and 2% antibiotic/antimycotic (all from Sigma-Aldrich). Cultures (5 × 10^6^ cells/mL) were stimulated with *C. tropicalis* yeasts killed by heat and pressure (HK-*Ct*) at a 5:1 ratio (five yeasts for each cell) or MOG (20 µg/mL) and incubated at 37 °C and 5% CO_2_ for 48 h. Supernatants were stored at −80 °C for subsequent cytokine quantification.

### 2.8. Cytokine Quantification

Quantification of interferon-gamma (IFN-γ), tumor necrosis factor-alpha (TNF-α), interleukin (IL)-2, IL-6, IL-10, and IL-17 were performed by enzyme-linked immunosorbent assay (ELISA) according to the manufacturers’ Set Kit (R&D Systems, Minneapolis, MI, USA).

### 2.9. Histopathological Evaluation

Lumbar spinal cord and kidney samples were fixed in 10% neutral buffered formalin (24 h), washed in water for 16–18 h, and immersed in 70% ethyl alcohol. The samples were then dehydrated in series of absolute ethyl alcohol and xylol and embedded in Paraplast Plus (Sigma-Aldrich). Four µm-thick histological sections were stained with HE (Hematoxylin-Eosin) for analysis of inflammatory infiltrate, LFB (Luxol Fast Blue) for demyelinization evaluation, and PAS (Periodic acid-reactive Schiff) for yeast detection, and then analyzed under an optical microscope. A semi-quantitative analysis of CNS inflammation was performed according to the following criteria: 0—inflammatory infiltration absent; 1—mild inflammatory infiltration; 2—moderate inflammatory infiltration, and 3—intense inflammatory infiltration. Besides the presence of inflammatory cells, kidney sections were also analyzed concerning edema, necrosis, and the presence of urinary casts. Altogether, these alterations were evaluated considering the following score system: 0—absence of renal parenchyma involvement; 1—discreet (<25%) involvement; 2—moderate (26–50%) involvement; 3—intense (>50%).

### 2.10. Isolation of CNS Mononuclear Cells

The brain and spinal cord were collected, macerated using sterile plastic pestles, and digested with 2.5 mg/mL collagenase D (Roche Applied Science, Indianapolis, IN, USA) and 100 μg/mL DNAse (Sigma-Aldrich) in 4 mL RPMI for 45 min at 37 °C. The suspensions were then washed in Hank’s Balanced Salt Solution (HBSS) by centrifugation at 450× *g*, 4 °C for 7 min, resuspended in 30% Percoll (GE Healthcare, Chicago, IL, USA), gently placed over 70% Percoll in 15 mL tubes, and then centrifuged at 950× *g*, 4 °C for 20 min with centrifuge brakes deactivated. After centrifugation, the ring containing mononuclear cells was collected, washed in RPMI, and centrifuged again at 450× *g*, 4 °C for 7 min. The cell pellet was then resuspended in flow cytometry staining buffer (PBS, 5% fetal bovine serum, and 0.1% NaN_3_) for flow cytometric analysis.

### 2.11. Flow Cytometry

CNS and kidneys isolated cell suspensions were adjusted to 5 × 10^5^ and 1 × 10^6^ cells/tube, respectively. Cells were labeled with the following fluorochrome-labeled antibodies (eBioscience, San Diego, CA, USA): 0.5 µg of FITC conjugated anti-CD45 (clone 30-F11); 0.14 µg of APC-eFluor 780 conjugated anti-CD11b (clone M1/70); 0.14 µg of PE-Cy7 conjugated anti-CD4 (clone GK1.5); 0.2 µg of APC conjugated anti-CD8 (clone 53–6.7) and 0.12 µg of PE-conjugated anti-Vβ11 (clone RR3–15) for 30 min at 4 °C. Data acquisition was performed using a FACSCanto II (BD Biosciences, San Jose, CA, USA) (IBB, UNESP, Botucatu, SP, Brazil), and the data were analyzed with FlowJo software (Becton Dickinson and Company, Franklin Lakes, NJ, USA). Flow cytometric gate strategy for leukocyte populations was described in Figure S1.

### 2.12. RT-PCR

Lumbar spinal cord and kidney samples frozen in liquid nitrogen were used for RNA extraction with Trizol reagent (Invitrogen, Carlsbad, CA, USA). cDNA synthesis (RT-PCR) was then performed according to the manufacturer’s recommendations (High-Capacity RNA-to-cDNA converter kit, Applied Biosystems, Foster city, CA, USA). The expression of T-box transcription factor/T-box expressed in T cells (*Tbx21/T-bet*) (Mm00450960_m1), GATA binding protein 3 (*GATA-3*) (Mm00484683_m1), RAR-related orphan receptor C (*Rorc/ROR-γt*) (Mm01261022_m1), forkhead box P3 (*Foxp3*) (Mm00475162_m1), and C-X3-C Motif Chemokine Receptor 1 (*CX3CR1*) (Mm00438354_m1) genes were analyzed based on the levels of Glyceraldehyde 3-phosphate dehydrogenase (*GAPDH*) (Mm99999915_m1) reference gene. Real-time PCR was performed using the Taqman system according to the manufacturer’s recommendations (Applied Biosystems). Gene expression was represented as relative fold change (2^−ΔΔCt^) using an uninfected and not submitted to the EAE induction group as a calibrator.

### 2.13. Statistical Analysis

Normality was tested using a Shapiro–Wilk test for all data. To compare the means of the 2 groups, a *t*-test was used in case of normal distribution. Otherwise, a gamma distribution was used in this comparison. Considering a 2-way layout, interaction means were compared using analysis of variance (ANOVA) followed by Tukey′s test in case of normality. If the distribution was asymmetric, a gamma distribution followed by Wald’s test was used. Survival data were evaluated using a Kaplan–Meier product limit estimate, and the log-rank test was used to compare the curves according to the groups. For count variables, a Poisson model followed by Wald’s test was used to compare means. Data were analyzed using SAS for Windows software, v.9.4, and values of *p* < 0.05 were considered statistically significant. Graphs and figures were made in GraphPad Prism 8 (GraphPad Software Inc., San Diego, CA, USA). Results were expressed as mean ± standard deviation (SD).

## 3. Results

### 3.1. Candida tropicalis Spreads to the CNS

**Figure 1:**
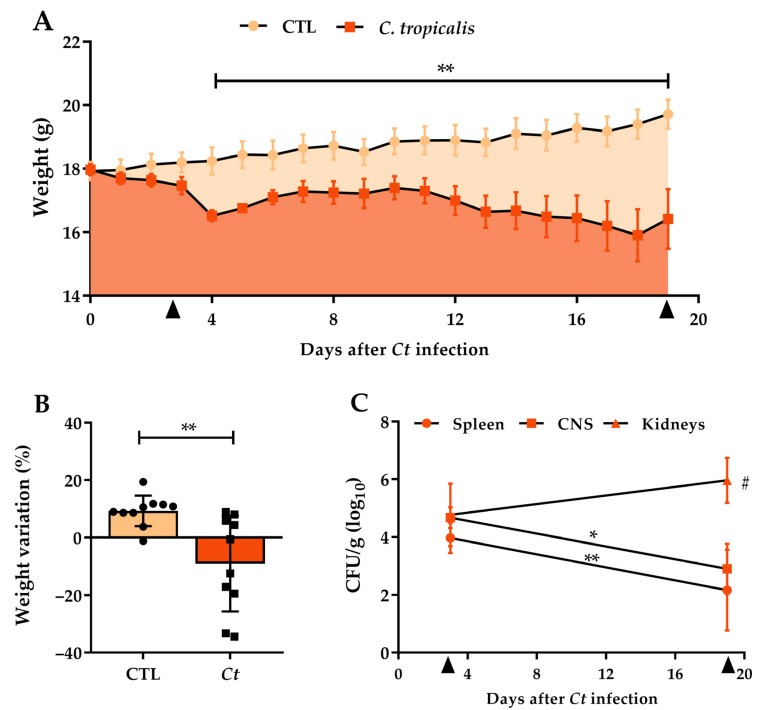
*Candida tropicalis* systemic infection in C57BL/6 mice. Female C57BL/6 mice were infected with 1 × 10^6^ viable *C. tropicalis* yeasts, and body weight was checked daily (**A**). The percentage of weight loss (**B**) was calculated considering the body weight variation between the initial and the final day of the experiment. The animals were evaluated on the 3rd and 19th days for the number of colony forming units per gram of tissue (CFU/g) in the CNS (brain and spinal cord), spleen and kidneys (**C**). The results are expressed as mean ± SD; *n* = 6/group (3 days); *n* = 10/group (19 days); two independent experiments; * *p* < 0.05 and ** *p* < 0.01. # Represent significant difference in CFU between kidneys and CNS/spleen samples at 19th day after infection.

The first stage of the study involved the characterization of *C. tropicalis* infection in female C57BL/6 mice. Initially, we used a 5 × 10^6^ yeasts/animal inoculum, which resulted in elevated weight loss and death of 33.3% of animals (data not shown). Subsequently, two smaller concentrations were tested: 2.5 × 10^6^ and 1 × 10^6^ yeasts/animal. Both determined much lower weight loss and smaller fungal loads comparing to the initial inoculum (Table 1). Thus, the smallest inoculum was chosen to carry out the study. Female C57BL/6 mice intravenously infected with 1 × 10^6^ viable *C. tropicalis* yeasts (*Ct* group) were evaluated 3 and 19 days after infection. A significant body weight loss was already noticed from the fourth day of infection in comparison to the non-infected group (CTL, control), as displayed in Figure 1A. The percentage of total body weight variation in these two experimental groups is illustrated in Figure 1B. The establishment of a widespread infection was confirmed by the recovery of colony-forming units (CFU) from the spleen, kidneys, and CNS at both evaluated moments. At the beginning of infection, no difference in the fungal load was observed among organs, but on the 19th day of infection, we observed a significantly higher fungal load in the kidneys compared to the spleen and CNS (Figure 1C). A comparative analysis between 3 and 19 days after infection revealed a decreased fungal load in the spleen and CNS while the fungal load in the kidneys remained similar (Figure 1C).

**Figure 2:**
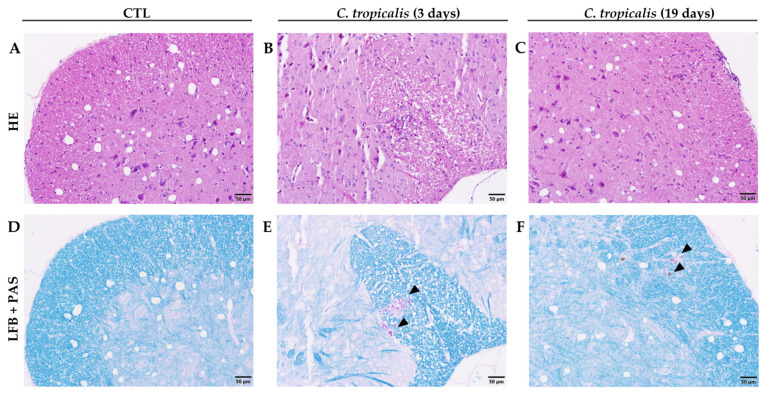
Dissemination of *C. tropicalis* to the spinal cord of C57BL/6 mice. Female C57BL/6 mice were infected with 1 × 10^6^ viable *C. tropicalis* yeasts and evaluated for inflammatory infiltrate (**A**–**C**) and presence of yeasts (**D**–**F**), indicated by arrows, in the lumbar spinal cord. Representative images of each group.

**Figure 3:**
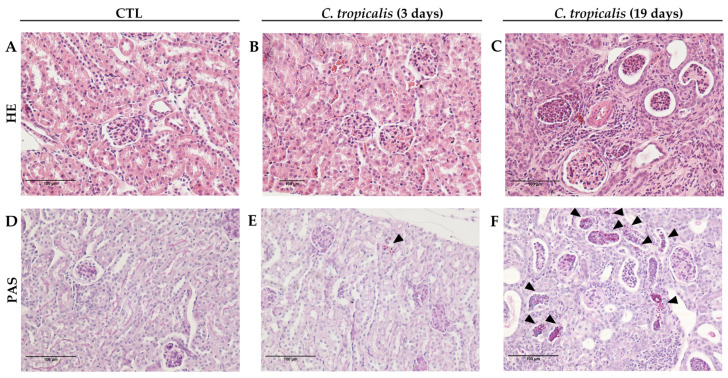
*C. tropicalis* growth within the kidneys of C57BL/6 mice. Female C57BL/6 mice were infected with 1 × 10^6^ viable *C. tropicalis* yeasts and evaluated for inflammatory infiltrate (**A**–**C**) and presence of yeasts (**D**–**F**), indicated by arrows, in the kidneys. Representative images of each group.

Although *C. tropicalis* had disseminated to the CNS (Figure 2E,F), the inflammatory process in the lumbar spinal cord samples was very discreet (Figure 2B,C), in comparison to CTL mice (Figure 2A,D). Interestingly, renal involvement worsened between days 3 and 19 after infection. Renal alterations were very discreet after 3 days of infection and were characterized by a few inflammatory aggregates containing fungus circumscribed by polymorphonuclear neutrophils and mononuclear cells (Figure 3B,E), in comparison to CTL mice (Figure 3A,D). Contrastingly, on the 19th day, all animals presented acute tubulointerstitial nephritis characterized by an intense inflammatory process. This process included an impressive infiltration of neutrophils and mononuclear cells, granular casts, edema, and areas of necrosis. Even though these lesions were mostly concentrated in the cortical areas, they frequently extended to the renal medulla (Figure 3C). Nevertheless, the presence of yeasts in the kidneys was associated with an intense inflammatory infiltration (Figure 3F), indicating a fungal tropism to kidneys after 19 days.

### 3.2. Cytokine Production in C. tropicalis Infected Mice

**Figure 4:**
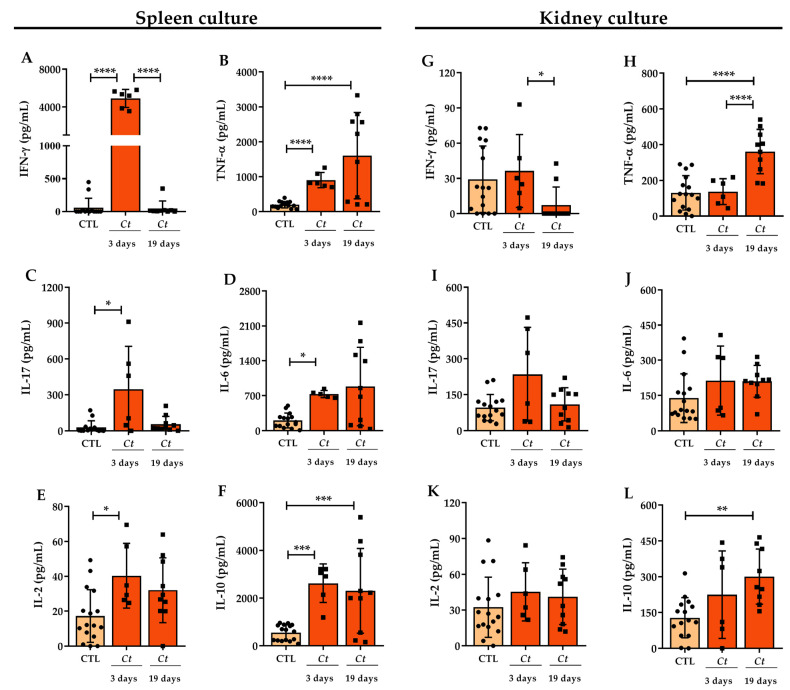
Cytokine production by splenic and renal cells of *C. tropicalis* infected mice. Female C57BL/6 mice were infected with 1 × 10^6^ viable *C. tropicalis* yeasts and evaluated after 3 and 19 days for cytokine production. IFN-γ, TNF-α, IL-17, IL-6, IL-2, and IL-10 levels were quantified in the total leukocyte culture supernatant of the spleen (**A**–**F**) and kidneys (**G**–**L**) stimulated with *C. tropicalis* yeasts killed by heat and pressure (25 × 10^6^/mL). The results are expressed as mean ± SD; *n* = 6/group (3 days); *n* = 10/group (19 days); two independent experiments; * *p* < 0.05; ** *p* < 0.01; *** *p* < 0.005, and **** *p* < 0.001.

To partially characterize the intensity of the immune response triggered by *C. tropicalis* systemic dissemination, the cytokine production was analyzed on the 3rd and 19th day after infection. For this purpose, cells isolated from the spleen and kidneys were cultured in the presence of dead *C. tropicalis* yeasts at a ratio of 5:1 (yeasts: cell). Concerning spleen cell cultures performed on the third day after infection, there was an expressive production of IFN-γ, TNF-α, IL-17, IL-6, IL-2, and IL-10, as shown in Figure 4. Alterations at the splenic cytokines, including decreased IFN-γ levels (Figure 4A) and increased TNF-α and IL-10 (Figure 4B,F), were observed after 19 days of infection. Concerning kidney cell cultures after three days of infection, no differences were observed in cytokine production by infected and control normal animals, as indicated in Figure 4. However, similarly to spleen cell cultures, decreased IFN-γ levels (Figure 4G) and increased TNF-α and IL-10 levels (Figure 4H,L) were observed in kidney cell cultures after 19 days of infection.

### 3.3. Effect of C. tropicalis Infection on EAE Development

Considering that *C. tropicalis* reached the lumbar spinal cord, induced the production of encephalitogenic cytokines in the periphery and that these cytokines could cross the blood–brain barrier [35,36], we presumed that *C. tropicalis* systemic infection could interfere in the CNS immune response. To assess the effect of *C. tropicalis* infection in the CNS, we used a murine model of encephalomyelitis (EAE), which was characterized by neuroinflammation and demyelination [18]. Female C57BL/6 mice intravenously infected with 1 × 10^6^ viable *C. tropicalis* yeasts were, after three days of infection, immunized with a myelin oligodendrocyte glycoprotein peptide (MOG_35–55_) to develop encephalomyelitis. We chose to induce the EAE on the third day of infection because on this timepoint there was fungus in the CNS and an elevated potential to produce cytokines. The animals were evaluated daily for clinical score and body weight until the 16th day after EAE induction, at which time other parameters were evaluated.

**Figure 5:**
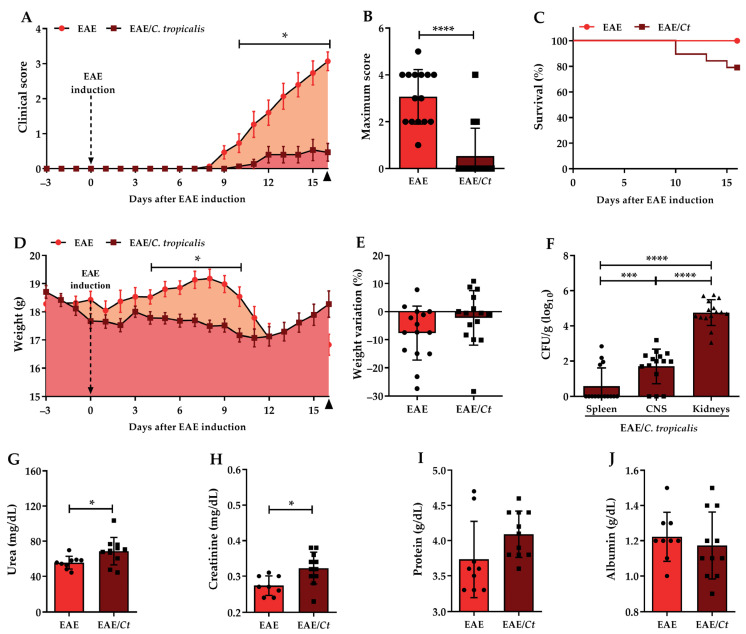
Effect of *C. tropicalis* infection on EAE development. Female C57BL/6 mice were infected with 1 × 10^6^ viable *C. tropicalis* yeasts three days before EAE induction and evaluated for the daily clinical score (**A**), maximum achieved score (**B**), survival percentage (**C**), daily body weight (**D**), and weight variation percentage considering the initial and the final weight (**E**). The number of colony forming units per gram of tissue (CFU/g) in spleen, CNS, and kidney (**F**) samples and levels of urea (**G**), creatinine (**H**), protein (**I**), and albumin (**J**) were evaluated on the 16th day after EAE induction. The results are expressed as mean ± SD; *n* = 15/group (**A**–**F**); *n* = 9–11/group (**G**–**J**); three independent experiments; * *p* < 0.05; *** *p* < 0.005, and **** *p* < 0.001.

Previously infected mice (EAE/*Ct* group) developed a clinically less severe paralysis (Figure 5A) or did not even develop the disease (Table 2). Considering all animals, sick and non-sick, a significant difference in the mean of maximum score between the EAE and EAE/*Ct* groups (Figure 5B) was observed. Although reducing or even blocking the development of EAE, *C. tropicalis* infection associated with EAE caused the death of some animals, resulting in a 78.9% survival rate (Figure 5C), whereas no death was observed in the EAE non-infected mice group. Regarding body weight, a significant loss was observed between the 4th and 10th days in the EAE/*Ct* group, but considering the initial and the final body weight, there was no significant difference between those two experimental groups (Figure 5D,E). Dead animal data were used only to define the survival; samples from these mice were not included in other analyses.

On the 16th day after EAE induction, the EAE/*Ct* group was also evaluated for fungal load both in the periphery (spleen and kidneys) and in the CNS. CFU were recovered from these three organs; however, the renal fungal load was significantly increased compared to both spleen and CNS (Figure 5F).

Analysis of biochemical parameters in serum samples indicated an increase in urea and creatinine levels in EAE/*Ct* group compared to the EAE group (Figure 5G,H), with no significant difference in total protein and albumin levels.

**Figure 6:**
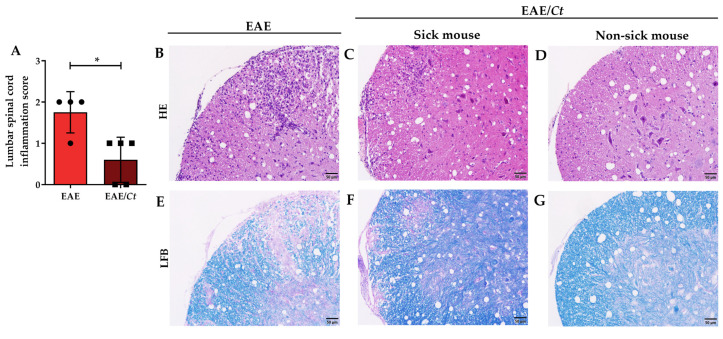
Effect of *C. tropicalis* infection on neuroinflammation in EAE mice. Female C57BL/6 mice were infected with 1 × 10^6^ viable *C. tropicalis* yeasts three days before EAE induction and evaluated for inflammatory infiltrate (**A**–**D**) and demyelination (**E**–**G**) in the lumbar spinal cord. Representative images of each group. The results are expressed as mean ± SD; * *p* < 0.05.

The lower severity of EAE in infected animals (EAE/*Ct* group) was confirmed by histopathological analysis of the lumbar spinal cord, which revealed less inflammatory infiltrate (Figure 6A–D) and less demyelization (Figure 6E–G).

**Figure 7:**
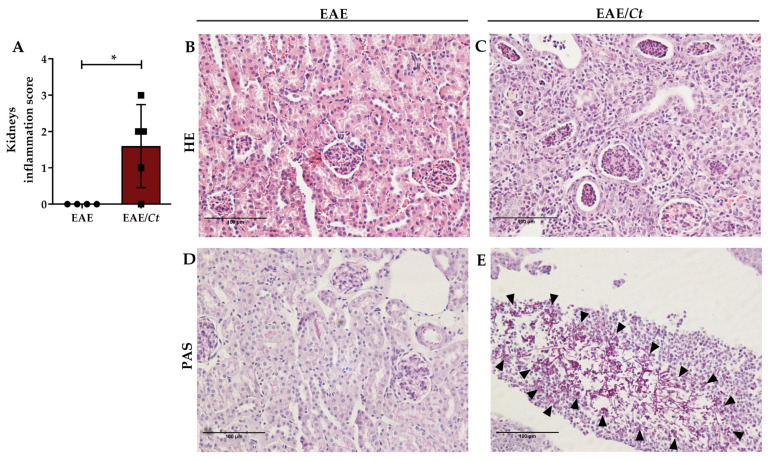
Effect of *C. tropicalis* infection on kidneys of EAE mice. Female C57BL/6 mice were infected with 1 × 10^6^ viable *C. tropicalis* yeasts three days before EAE induction and evaluated for renal parenchyma impairment (**A**–**C**) and presence of yeasts (**D**,**E**), indicated by arrows, in kidney samples. Representative images of each group. The results are expressed as mean ± SD; * *p* < 0.05.

In addition, this preferential accumulation of the fungus in the kidneys caused an accentuated local inflammation triggering acute tubulointerstitial nephritis (Figure 7).

### 3.4. C. tropicalis Downmodulates Peripheral Production of MOG-Induced IFN-γ and IL-2

**Figure 8:**
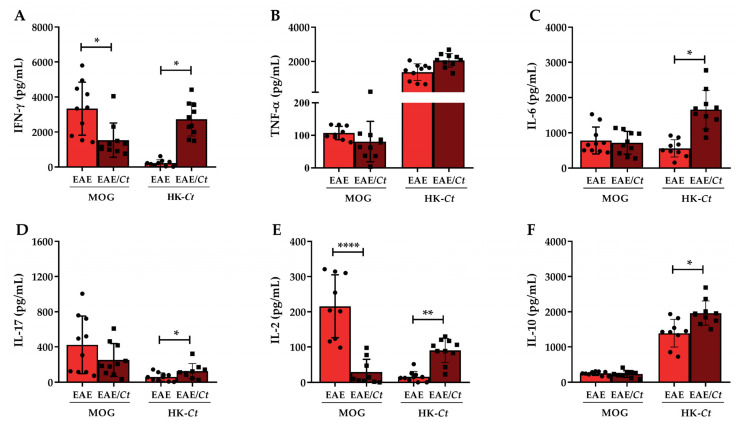
Cytokine production by splenic cells of EAE mice previously infected with *C. tropicalis*. Female C57BL/6 mice were infected with 1 × 10^6^ viable *C. tropicalis* yeasts three days before EAE induction and evaluated for cytokine production on the 16th day after EAE induction. IFN-γ (**A**), TNF-α (**B**), IL-6 (**C**), IL-17 (**D**), IL-2 (**E**), and IL-10 (**F**) levels were quantified in total leukocyte culture supernatant of spleen stimulated with *C. tropicalis* yeasts killed by heat and pressure (25 × 10^6^/mL) or stimulated with MOG_35–55_ (20 µg/mL). The results are expressed as mean ± SD; *n*= 10–11/group; two independent experiments; * *p* < 0.05; ** *p* < 0.01, and **** *p* < 0.001.

To investigate whether *C tropicalis* interference on EAE development included modulation of the peripheral immune response, total spleen cells were cultured in the presence of dead *C. tropicalis* yeasts (HK-*Ct*) or MOG, that was, infectious agent-specific antigens and neuro-antigen, respectively. As shown in Figure 8, splenic cells from the EAE/*Ct* group stimulated with HK-*Ct* produced significantly higher levels of IFN-γ, IL-6, IL-17, IL-2, and IL-10 than the EAE group. Cytokine production induced by MOG was quite similar in both groups except for IFN-γ and IL-2, whose levels were significantly lower in the EAE/*Ct* group (Figure 8A,E).

### 3.5. C. tropicalis Diverts Leukocyte Infiltration to the Kidneys

**Figure 9:**
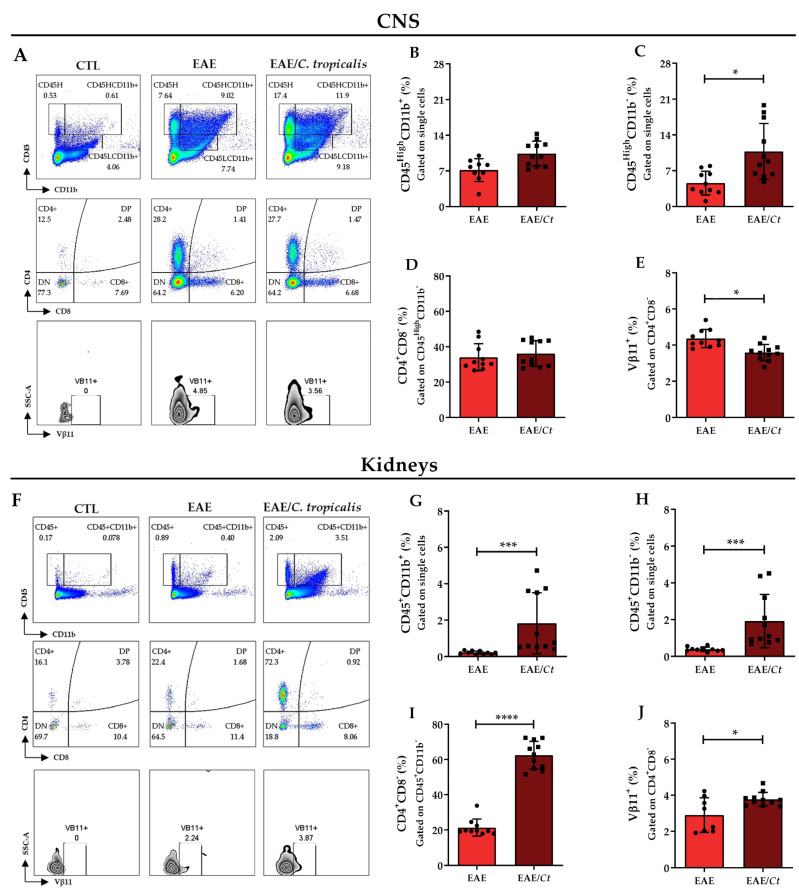
Leukocyte subpopulations in the CNS and kidneys of EAE and EAE/Ct mice. Female C57BL/6 mice were infected with 1 × 10^6^ viable *C. tropicalis* yeasts three days before EAE induction and evaluated for the percentage of myeloid cells (CD45^High^CD11b^+^), other immune cells (CD45^High^CD11b^−^), CD4^+^ cells (CD4^+^CD8^−^), and TCR expression (Vβ11^+^) in the CNS (**A**–**E**) and kidneys (**F**–**J**) on the 16th day after EAE induction. Representative flow cytometric plots for leukocyte populations in the CNS (**A**) and kidneys (**F**). The results are expressed as mean ± SD; *n* = 10–11/group; two independent experiments; * *p* < 0.05; *** *p* < 0.005, and **** *p* < 0.001.

Considering the CNS and kidneys as the main sites of injury associated with EAE and the infection by *C. tropicalis*, respectively, we evaluated the presence of immune response effector cells in these tissues by flow cytometry (Figure S1). To assess leukocyte infiltration in the CNS (Figure 9A), we analyzed CD45^High^ plus the expression or not of CD11b in single cells. CD45^High^ population that expressed CD11b was considered to be constituted of macrophages or activated microglia, whereas CD45^High^ population that did not express CD11b was considered to represent other immune cells [37]. In the kidneys, CD45^+^ cells were evaluated that expressed or did not express CD11b (Figure 9F). The percentage of these leukocyte populations was significantly increased in the CNS (Figure 9C) and in the kidneys (Figure 9G,H) from the EAE/*Ct* group compared to the EAE group. Within the CD45^High^CD11b^−^ population, the percentage of CD4^+^CD8^−^ cells was significantly higher in the kidneys of EAE/*Ct* group compared to the EAE group (Figure 9I), with no difference in the CNS (Figure 9D) and in the percentage of CD4^−^CD8^+^ T lymphocytes in both organs (data not shown). Next, we evaluated the usage of the T-cell receptor (TCR) beta-chain Vβ11^+^. This specific TCR beta-chain was expressed in C57BL/6 mice and its increased usage in T cells was demonstrated in response to both *M. tuberculosis* [38] and MOG [39], whose antigens were present in the emulsion applied to C57BL/6 mice to induce EAE. Although we did not provide information about the antigen specificity of these cells to *M. tuberculosis* or MOG, our analysis of CD4^+^CD8^−^Vβ11^+^ T cells revealed an inversely proportional relationship, with a decrease of this population in the CNS (Figure 9E) and an increase in the kidneys (Figure 9J) of animals from the EAE/*Ct* group.

**Figure 10:**
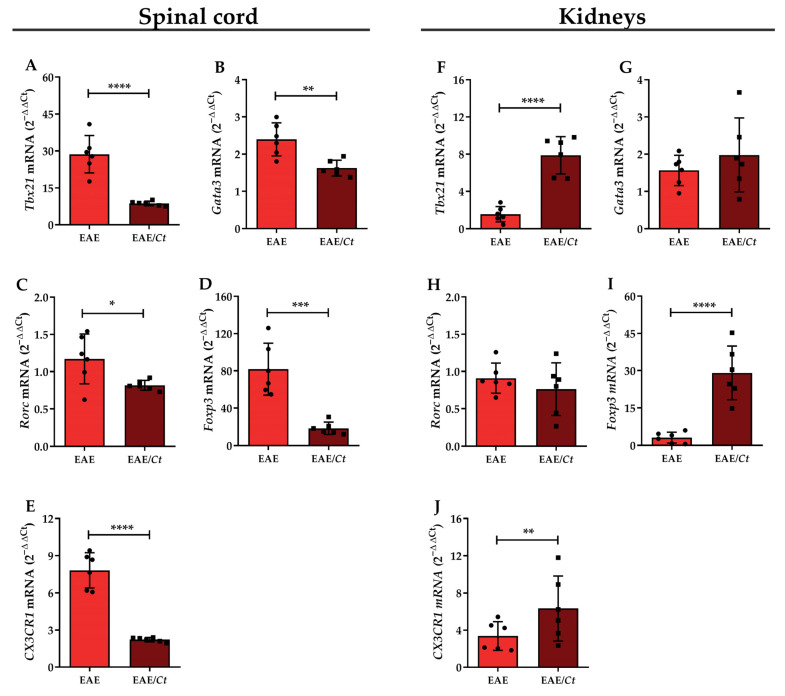
T lymphocyte subpopulations in EAE mice. Female C57BL/6 mice were infected with 1 × 10^6^ viable *C. tropicalis* yeasts three days before EAE induction and evaluated for relative expression of *Tbx21*, *Gata3*, *Rorc*, *Foxp3*, and *CX3CR1* mRNA in the lumbar cord (**A**–**E**) and kidney (**F**–**J**) homogenate on the 16th day after EAE induction. The results are expressed as mean ± SD; *n* = 6/group, one representative experiment of two; * *p* < 0.05; ** *p* < 0.01; *** *p* < 0.005, and **** *p* < 0.001.

Additionally, the expression of transcription factors associated with T helper (Th) 1, Th2, Th17, and regulatory T cells (Treg) was evaluated in the lumbar spinal cord and kidneys. A significant decrease was observed in the relative mRNA expression of *Tbx21* (Th1), *Gata3* (Th2), *Rorc* (Th17), and *Foxp3* (Treg) genes in the lumbar spinal cord of the EAE/*Ct* group compared to the EAE group (Figure 10A–D). Interestingly, an inversion in the relative mRNA expression pattern of the *Tbx21* (Th1) and *Foxp3* (Treg) genes was observed in the kidneys, with significantly higher expression in the EAE/*Ct* group than in the EAE group (Figure 10F,I). The relative mRNA expression of the *CX3CR1* gene, a chemokine receptor, was significantly decreased in the lumbar spinal cord and increased in the kidneys of EAE/*Ct* group (Figure 10E,J). The *GAPDH* gene was used as an endogenous control since its expression was similar among the groups (Figure S2).

## 4. Discussion

Multiple sclerosis (MS) is considered the most relevant cause of disability in young adults [40], and it is well established that its development can be affected by different environmental factors, including infectious agents. Animal models, especially experimental autoimmune encephalomyelitis (EAE) induced in mice, have been substantially used to investigate the immunopathogenesis of this disease [17,18,41]. By using a murine model, we examined the effect of *Candida tropicalis* infection, considered the second most virulent species of the genus *Candida* [9], in the evolution of EAE.

Initial experiments indicated, as expected that the severity of *C. tropicalis* infection in C57BL/6 mice is inoculum-dependent. The highest concentration (5 × 10^6^) injected by the lateral tail vein determined high mortality and, therefore, lower inocula were tested. These experiments indicated that intravenous infection with 1 × 10^6^ viable yeasts was not lethal for C57BL/6 mice and resulted in dissemination to vital organs, including the brain and spinal cord. This was, therefore, the chosen concentration to test the effect of a previous infection with this fungus on EAE development.

Although not lethal, the infection determined body weight loss from the fourth day of infection. Analogously to *C. albicans* [27], this fungus was able to invade both the brain and spinal cord. As expected, the fungus disseminated through the spleen and kidneys, which are relevant organs in terms of immune response and body homeostasis, respectively. Although the fungal load was diminished in the spleen and the CNS after two weeks of infection, it remained elevated in the kidneys, suggesting a tropism of the *C. tropicalis* for this organ. The kidney involvement by *C. tropicalis* was already described in a murine experimental model [42] and in humans [15,43], which was relevant in most systemic *Candida* infections since this may lead to death by renal failure [44].

Considering the adoption of a systemic infection, we initially determined the profile of cytokines produced by spleen cells and inflammatory cells retained in the kidneys. The splenic profile included high production of proinflammatory mediators such as IFN-γ, TNF-α, IL-6, and IL-2, as well as high levels of the anti-inflammatory IL-10. The presence of proinflammatory cytokines is fundamental to remove the fungus through phagocyte activation [45]. On the other hand, the relevance and complexity of IL-10 rely on the fact that despite its putative homeostatic role in controlling inflammation [46,47], this cytokine could also interfere with the effectiveness of protective immunity, leading to fungal persistence [1,48]. Interestingly, the protective or detrimental effects of IL-10 depended upon the degree of inflammation in an experimental candidiasis model [49]. In this context, IL-10 might be required to limit tissue damage under highly inflammatory circumstances [50]. The observed increase in IL-2 and IL-10 production, concomitantly with a reduction in IFN-γ and IL-17 levels on the 19th day of infection, could indicate expansion of Treg cells as already described in response to *C. albicans* [48].

Kidney cell cultures produced high levels of TNF-α and IL-10 but not IFN-γ, IL-17, and IL-2, suggesting that the cytokine cell source was predominantly from the innate immunity, probably phagocytes [51,52]. Phagocytes present in kidneys during *Candida* infections, including macrophages and neutrophils, are associated with fungal clearance and resolution of infection [53]. However, the renal inflammatory process may result in tissue damage and consequently affect the proper functioning of the organ [54]. In our study, a high fungal load was maintained in the kidneys even in the presence of pro-inflammatory cytokines, indicating that the immune response in the kidneys did not control the infection.

The presence of the fungus for a long period in the kidneys could act as a source of fungal antigens and promote a constant activation of the immune system. In this scenario, cytokines produced in the periphery could cross the blood–brain barrier and thus contribute to the inflammatory process in the CNS [35,36]. Moreover, the presence of *C. tropicalis* in the CNS itself could directly activate the local immune response. This scenario reinforced our initial hypothesis that *C. tropicalis* present in the CNS or in a non-neurological site, could interfere in the EAE development by initiating or even exacerbating a neuroinflammatory process.

We then evaluated the effect of this non-*albicans Candida* species on the evolution of EAE. For this, the animals were infected with *C. tropicalis* and, on the third day after infection, were submitted to EAE induction. Surprisingly, infected mice were partially protected from encephalomyelitis; both prevalence and severity of the disease were lower in this experimental group. This finding was initially interpreted as protection, however, the survival rate of infected animals was lower compared to uninfected EAE animals. Two possibilities were then considered from this result: whether the infection was inducing a CNS regulatory response or diverting the CNS immune response to a non-neurological site, such as the kidneys.

Microbiological analysis showed that the fungus had spread to the CNS, spleen, and kidneys, being the highest fungal load found in the kidneys, even with the presence of hyphal forms. Some biochemical tests were then performed to evaluate a possible renal impairment. The higher levels of urea and creatinine in the serum of infected animals confirmed that *C. tropicalis* affected the renal function in EAE mice. In a similar way, Louria and colleagues demonstrated that systemic *C. tropicalis* infection in mice was associated with rupture into and growth within the renal tubular lumen and transient multiplication in the brain [34]. Although not directly related to *Candida* infections, kidney infection was considerably higher in MS compared with non-MS patients [55,56] and associated with a worse disease prognosis [57].

To investigate the mechanism by which *C. tropicalis* infection reduced EAE severity, we initially checked cytokine production by spleen and kidney cell cultures from these animals. Both cultures were stimulated with heat-killed *C. tropicalis* yeast (HK-*Ct*) (fungus-specific particulate antigen) and with MOG (CNS-derived soluble antigen). As expected, the stimulation with HK-*Ct* determined elevated production of IFN-γ, IL-6, IL-17, IL-2, and IL-10 in the EAE/*Ct* group. Noteworthy, these pro-inflammatory cytokines are involved in immune responses against *Candida* spp. and in the immunopathogenesis of MS [1,45,58]. On the other hand, cytokine quantification in cultures stimulated with MOG revealed an interesting fact characterized by lower production of IFN-γ and IL-2 in the cultures from animals of the EAE/*Ct* group, suggesting that the presence of the fungus was, in some way, affecting the MOG-specific immune response. These results indicate, as expected that previously infected EAE mice respond to both infection-specific antigens and neuro-antigen, whereas EAE non-infected mice respond only to neuro-antigen, supporting the hypothesis of a pro-inflammatory effect of the fungus.

Histopathological analysis showed lower inflammatory infiltrate and demyelination in the lumbar spinal cord of EAE infected mice, which reinforces the idea that inflammation could be diverted to the periphery. Considering this lower CNS involvement and the synchronic higher kidney involvement, additional assays were performed to investigate further the presence of certain cell populations, especially CD4^+^ cells that express Vβ11^+^. CD4^+^ cells derived from C57BL/6 mice immunized with MOG_35–55_ peptide emulsified in CFA and restimulated in vitro with MOG_35–55_ expressed a TCR composed of Vα3.2 and Vβ11. This cell clone expanded in vitro, called 2D2, gave rise to transgenic mice with myelin-reactive T cells [39], representing the encephalitogenic T lymphocytes [59]. Flow cytometric analysis revealed that the proportion of CD45^+^CD11b^−^ cells were higher in the CNS of the EAE/*Ct* group, whose encephalomyelitis was less severe. Despite this, the proportion of CD4^+^Vβ11^+^ cells were significantly lower in the CNS and higher in the kidneys of these mice. Although in an indirect way, because we evaluated Vβ11 alone, nor in combination with Vα3.2, our result suggests that specific T lymphocyte clones were, at least partially, retained in the kidneys. In a similar way, the deviation of T lymphocytes from the CNS to peripheral inflammatory sites was described as a mechanism of mycobacteria-mediated suppression in EAE development [60].

The possible retention of lymphocytes in the kidney was reinforced when the lumbar spinal cord, the major target of lesions in the EAE [61], and kidneys were evaluated by real-time PCR to determine the mRNA expression of *Tbx21* (Th1), *Gata3* (Th2), *Rorc* (Th17), *Foxp3* (Treg), and *CX3CR1*. *CX3CR1* is a chemokine receptor found in resident macrophages and is essential for the anti-*Candida* response in mice, mediating the survival of these cells through inhibition of caspase-dependent apoptosis [62]. The expression level of all these genes was decreased in the spinal cord of the EAE/*Ct* group animals, a result consistent with the lower degree of lesions in these animals. On the other hand, except *Gata3*, the expression of the other genes was increased in the kidney of animals of this group, thus confirming that part of the leukocytes was retained in the kidneys.

Considering therapeutic possibilities, this result cannot be directly translated to patients with MS, since renal damage was severe and not acceptable. However, the result itself is proof of concept that inflammatory T cell retention strategies in the periphery can potentially prevent the migration of these cells to the CNS, thus preventing the worsening of this disease.

## 5. Conclusions

*C. tropicalis* infection reduces the prevalence and severity of EAE. The results suggest that this effect is due, at least partially, to the sequestration of leukocytes by the inflamed renal tissue. We conclude, therefore, that *C. tropicalis* infection reduces the development of EAE, but this effect is associated with severe involvement of renal function and may lead to death.

## Figures and Tables

**Table 1:** Adjustment of inoculum concentration. Female C57BL/6 mice were infected with 2.5 × 10^6^ and 1 × 10^6^ viable *C. tropicalis* yeasts and then compared for weight variation and fungal load.

Inoculum Concentration	Weight Variation (%)	Total Fungal Load (CFU/g log_10_)
2.5 × 10^6^ viable yeasts	−15.25 ± 2.27	2.99 ± 1.39
1 × 10^6^ viable yeasts	−8.82 ± 4.21	2.76 ± 1.26

**Table 2:** EAE incidence. Female C57BL/6 mice were infected with 1 × 10^6^ viable *C. tropicalis* yeasts three days before EAE induction and evaluated for the incidence of EAE until the 16th day after EAE induction.

Groups	Affected/Total	Incidence (%)
**EAE**	15/15	100%
**EAE/*C. tropicalis***	3/15	20%

## Data Availability

The data presented in this study are available on request from the corresponding author.

## Supplementary Materials

These data are available online at https://www.mdpi.com/article/10.3390/jof7090757/s1, Figure S1: Flow cytometric gate strategy for leukocyte populations; Figure S2: Expression of endogenous PCR control.
