# Supplementary material for: Candida tropicalis Systemic Infection Redirects Leukocyte Infiltration to the Kidneys Attenuating Encephalomyelitis
Source: J Fungi (Basel). 2021 Sep 14;7(9):757. doi: 10.3390/jof7090757 (PMC8471291; doi:10.3390/jof7090757)
Supplement: Supplementary file 1 [file jof-07-00757-s001.zip › jof-1361837-supplementary.pdf]

Journal of *Fungi*

MDPI

Supplementary Material

Candida tropicalis Systemic Infection Redirects Leukocyte Infiltration to the Kidneys Attenuating Encephalomyelitis

Natália Munhoz-Alves [1], Luiza Ayumi Nishiyama Mimura [1], Rosa Marlene Viero [2], Eduardo Bagagli [1], Jean Pierre Schatzmann Peron [3], Alexandrina Sartori [1,4], and Thais Fernanda de Campos Fraga-Silva [1]*

[1] Department of Chemistry and Biological Sciences, Institute of Biosciences, São Paulo State University (UNESP), Botucatu 18618-689, Brazil; natalia.mnhz@gmail.com (N.M.-A.); luizamimura@gmail.com (L.A.N.M.);
eduardo.bagagli@unesp.br (E.B.); alexandrina.sartori@unesp.br (A.S.)

[2] Department of Pathology, Botucatu Medical School, São Paulo State University (UNESP), Botucatu 18618-687, Brazil; rosa.viero@unesp.br

[3] Neuroimmune Interactions Laboratory, Department of Immunology, Institute of Biomedical Sciences (ICB) IV, University of São Paulo (USP), São Paulo 05508-000, Brazil; jeanpierre@usp.br

[4] Postgraduate Program in Tropical Diseases, Botucatu Medical School, São Paulo State University (UNESP), Botucatu 18618-687, Brazil

* Correspondence: thaisfragasilva@gmail.com

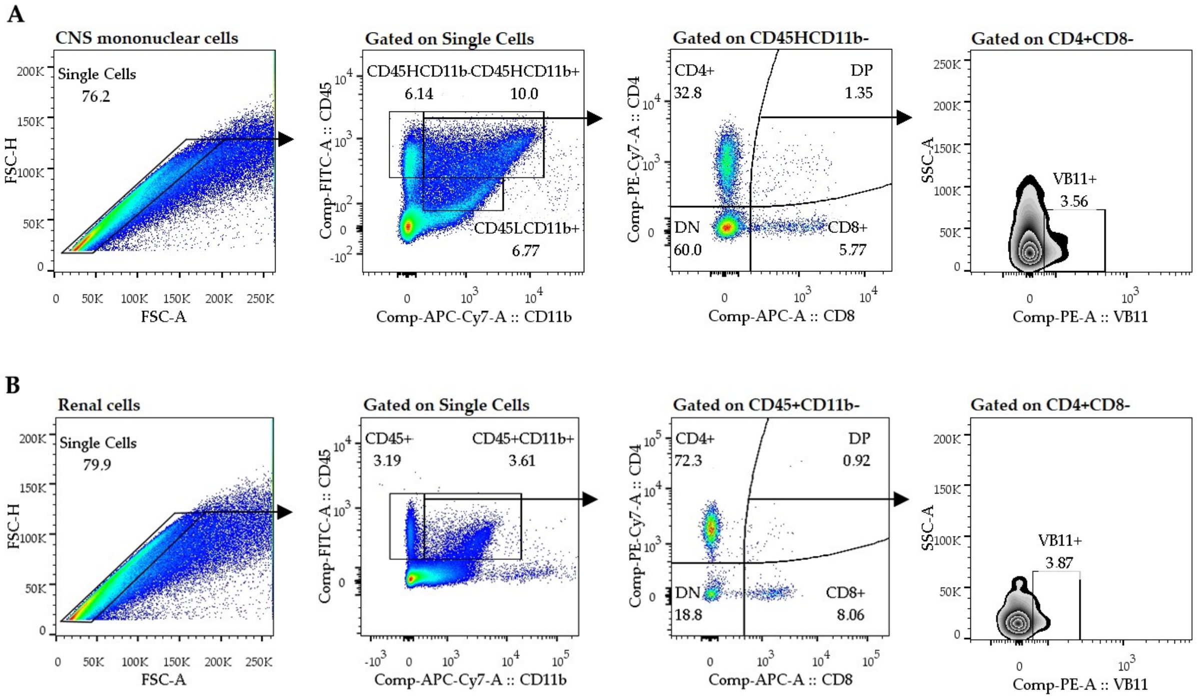

Figure S1. Flow cytometric gate strategy for leukocyte populations. CNS mononuclear cells (**A**) and renal cells (**B**) were collected and analyzed by flow cytometry. The percentage of macrophages or activated microglia ($CD45^{High}CD11b^{+}$) and other immune cells ($CD45^{High}CD11b^{-}$) was evaluated in single cells (FSC-H vs FSC-A) gating. The percentage of T helper lymphocytes ($CD4^{+}CD8^{-}$) was gating to assess the percentage of $VB11^{+}$ cells. Non-stained cells were used to define gating strategy. Flow cytometry images were created using FlowJo 10.7.1.

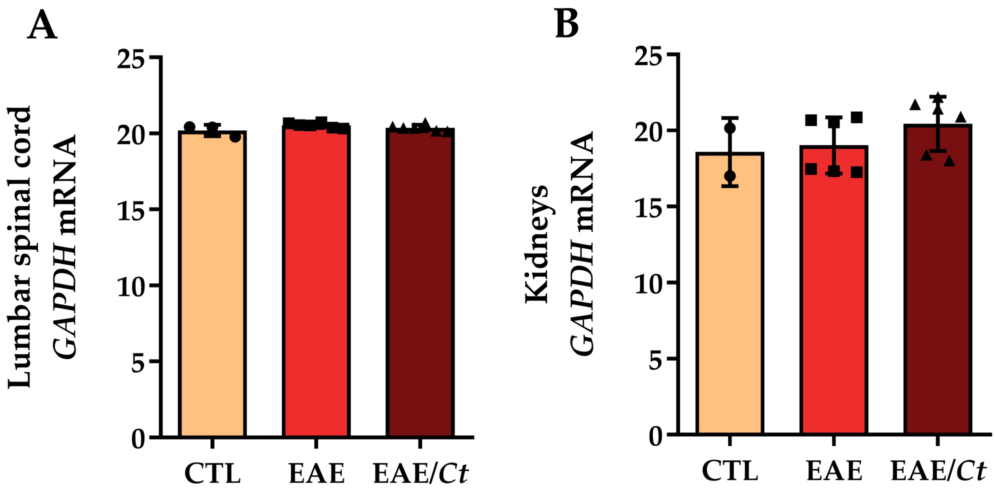

Figure S2. Expression of endogenous PCR control. Female C57BL/6 mice were infected with 1×10[6] viable *C. tropicalis* yeasts three days after EAE induction and evaluated for the *GAPDH* mRNA expression in lumbar spinal cord (**A**) and kidney (**B**) homogenate. The results are expressed as mean ± SD; n=2-3/group in CTL mice and n= 6/group in EAE mice.
